# Continuous intravenous infusion of remifentanil improves the experience of parturient undergoing repeated cesarean section under epidural anesthesia, a prospective, randomized study

**DOI:** 10.1186/s12871-019-0900-x

**Published:** 2019-12-30

**Authors:** Wei Yan, Yun Xiong, Yu Yao, Feng-jiang Zhang, Li-na Yu, Min Yan

**Affiliations:** 1 Department of Anesthesiology, Second Affiliated Hospital, School of Medicine, Zhejiang University, Zhejiang Province, China; 2 Huzhou Maternity & Child Health Care Hospital, Huzhou, Zhejiang Province, China

**Keywords:** Remifentanil, Epidural anesthesia, Repeated cesarean delivery

## Abstract

**Background:**

Unsatisfactory analgesia would occur frequently during repeated cesarean section under epidural anesthesia. The aim of this study is to observe the effects of intravenous remifentanil on maternal comfort, maternal and neonatal safety during repeated cesarean section under epidural anesthesia.

**Methods:**

A total of 80 parturients undergoing repeated cesarean section were involved in the study. The patients were randomly divided into the intravenous remifentanil- assisted epidural group (group R) and epidural group (group E), respectively (*n* = 40). In group R, the remifentanil was continuously intravenously infused as an adjuvant to epidural anesthesia. In group E, 0.75% ropivacaine epidural or intravenous ketamine was administered as needed. Parturient baseline characteristics, vital signs, VAS scores, and comfort scores during surgery were recorded. Adverse effects were also recorded.

**Results:**

A total of 80 patients were enrolled in the current study and the final analyses included 39 patients in group R and 38 patients in group E. No differences in patients’ baseline characteristics were found between the two groups (*p >* 0.05). Compared with group E, the comfort score was significantly higher in group R (9.1 ± 1.0 vs. 7.5 ± 1.3, *p <  0.001*), whereas the maximum VAS score was significantly lower in group R (1.8 ± 1.2 vs. 4.1 ± 1.0, *p <  0.001*). Maternal and neonatal adverse effects did not differ between the two groups during surgery (*p* > 0.05).

**Conclusions:**

Continuous intravenous infusion of low-dose remifentanil can significantly improve the experience of parturients undergoing repeated cesarean section under epidural anesthesia, without noticeable maternal or neonatal adverse effects.

**Trial registration:**

This study was pre-registered at http://www.chictr.org.cn/index.aspx (ChiCTR1800018423) on 17/09/2018.

## Background

Epidural anesthesia is a popular and safe anesthetic technique for cesarean section [1, 2], which has few maternal and neonatal adverse effects, as well as excellent controllability. However, visceral pain caused by visceral traction frequently occurs in parturient with epidural anesthesia during cesarean section [3, 4]. Additionally, when the anxiety regarding surgery is included, epidural anesthesia must not be a pleasant experience for parturients.

In China, with the implementation of the following child policy, the number of parturients with a scarred uterus has dramatically increased; this event leads to an increased number of intra-abdominal adhesions [5]. Parturients with a scarred uterus may experience longer surgery duration and higher intensity of peritoneal traction, compared with uniparous women [3], which results in more serious intraoperative visceral pain during cesarean section. Propofol, thiopentone, and ketamine have been administered intravenously as rescue analgesia during cesarean section; however, these may reduce the umbilical arterial pO_2_ and Apgar score of neonates [6, 7].

Whilst remifentanil is an off label drug for parturients and some serious adverse effects with remifentanil have been reported previously, it has been recently said that remifentanil is effective, has less adverse effects compared to other opioid medications and is safe to use in controlled circumstances [8–10]. In our clinic, we found that continuous intravenous infusion of low-dose remifentanil has a good rescue analgesic effect on incomplete epidural analgesia and provides a degree of sedation during cesarean section, which improves the patient experience. Thus, we designed this prospective, randomized, controlled study to investigate the effects of continuous intravenous infusion of remifentanil 0.05 μg·kg^− 1^·min^− 1^ on parturient experience and neonatal safety among patients undergoing epidural anesthesia during repeated cesarean section.

## Methods

### Ethics

The study was approved by the ethics committee of Huzhou Maternity & Child Health Care Hospital (Ethical Committee number 201801; Chairperson Ping-ya He) and written informed consent was provided by all patients before enrollment in the study. Our study adheres to CONSORT guidelines.

### Study design and patient population

A total of 80 patients with repeated cesarean section, aged 23–36 years (weight 56–90 kg, American Society of Anesthesiologists levels I and II) were involved in the study. The patients were randomly placed in either the intravenous remifentanil-assisted epidural group (group R) or the epidural group (group E).

### Criteria for inclusion and exclusion

Patients undergoing repeated cesarean section, with full-term, singleton pregnancies, who had arranged for epidural anesthesia, were included in this study. Patients with contraindications for epidural anesthesia, history of allergy to bupivacaine or opioids, history of spinal surgery, or intrauterine hypoxia were excluded from this study. Patients with epidural anesthesia puncture failure, poor effect of epidural anesthesia, or intraoperative hemorrhage were also excluded from the analysis.

### Preoperative preparations and anesthesia protocol

All patients fasted for 6 h and discontinued fluid intake 2 h before repeated cesarean section. Intravenous access was established, 5 L/min oxygen was administered, and Ringer’s lactate 8 ml/kg was preloaded after patients entered the operating room. Electrocardiography, noninvasive arterial pressure, respiratory rate, and pulse oximetry were routinely monitored for all parturients. RR was measured by carbon dioxide sampling.

Before epidural anesthesia was administered, patients were placed in the left lateral decubitus position. The epidural space was cannulated at the L2–3 interspace with the midline approach, using an 18-gauge Tuohy needle. A loss-of-resistance to the saline technique was used to affirm the puncture; then, a 20-gauge epidural catheter was advanced 3 cm cephalad. Three milliliters 1.5% lidocaine with 10 μg adrenaline was injected through the epidural catheter as a test dose. Epidural administration of 0.75% ropivacaine was performed in two groups until the epidural anesthesia level of all parturients reached T6. In group R, remifentanil was continuously intravenously infused at a rate of 0.05 μg·kg^− 1^·min^− 1^ at the beginning of the operation. The intravenous infusion rate of remifentanil was increased by 0.025 μg·kg^− 1^·min^− 1^ if patients complained of discomfort or pain, and the next rate of 0.025 μg·kg^− 1^·min^− 1^ would increase if the discomfort or pain was not relieved after 5 min. The maximum rate of intravenous infusion did not exceed 0.15 μg·kg^− 1^·min^− 1^. If excessive sedation or respiratory depression occurred, intravenous infusion of remifentanil was reduced by 0.025 μg·kg^− 1^·min^− 1^ until infusion was completely discontinued. In group E, if the patients complained of discomfort or pain, 0.75% epidural ropivacaine was administered as needed. Intravenous infusion of ketamine was administered as required (0.5 mg/kg per dose, repeated for 20 min if necessary) or general anesthesia was performed in both groups if the discomfort or pain were not relieved.

If the saturation of pulse oxygen (SpO_2_) was< 95% or respiratory rate (RR) was< 8 times/min (i.e., respiratory depression was observed), the parturient was awakened, and assisted breathing was applied. If the heart rate (HR) was < 50 beats/min, intravenous atropine 0.5 mg was administered. If hypotension (a systolic blood pressure (SBP) reduction of > 30% or a value of < 90 mmHg) occurred, intravenous ephedrine 5–10 mg was administered. All anesthesia procedures were performed by the same senior anesthesiologist, and all data were recorded by an anesthesia nurse. Patients in both groups were excluded from this study if the anesthetic block level did not reach T10, or if they were changed to general anesthesia 15 min after epidural administration.

### Measurements

The following parturient data were recorded: age, body mass index (BMI), weight, ASA status, dose of ropivacaine, gestational weeks, dose of remifentanil, and epidural anesthesia block level (counted from the sacral vertebra [11]) were recorded. SpO_2_, mean arterial pressure (MAP), HR, and RR were recorded before anesthesia (T_0_), as well as at skin incision (T_1_), delivery of baby (T_2_), uterine suture (T_3_), and intraoperative traction (T_4_) in all parturients. The visual analogue scale (VAS) score was recorded at T_1_, T_2_, T_3_, and T_4_; the maximum VAS score during surgery was also recorded. The level of sedation (evaluated by the Ramsay Sedation Scale) was recorded at T_0_, T_1_, T_2_, T_3_, and T_4_. The degree of comfort during surgery was assessed using the numerical rating scale (NRS, 0 = least comfort imaginable, 10 = very comfortable) [12]. The use of intraoperative oxytocin was recorded in both groups. Incidences of intraoperative respiratory depression (RR < 8 times/min), bradycardia, hypotension, and postoperative adverse reactions were recorded for both groups; Apgar scores were recorded at 1and 5 min after birth for both groups, as were the numbers of neonatal resuscitations and the pH value of neonatal umbilical arterial blood.

The comfort scores during surgery were the primary outcome measure of the study; the secondary outcomes were the maximum VAS score, and maternal and newborn adverse effects during surgery. In current study, another anesthesiologist was responsible for the data record.

### Statistical analysis

In a preliminary trial of 20 patients, the comfort scores during surgery were 8.9 ± 0.9 and 7.8 ± 1.6 in groups R and E, respectively. Based on the preliminary trial, 26 patients were required per group to detect a 15% increase of comfort score for 90% power and an α level of 0.05, with a drop-out rate of 15%. Data are expressed as mean ± standard deviation (SD), or numbers of patients, as appropriate. Statistical analyses were performed using independent t-tests, the chi-squared test, Fisher’s exact test, and repeated measures analysis of variance, as appropriate. All statistical analyses were performed using SPSS version 22.0 (SPSS Inc., Chicago, IL, USA). *p* <  0.05 was considered to indicate statistical significance.

## Results

**Fig. 1:**
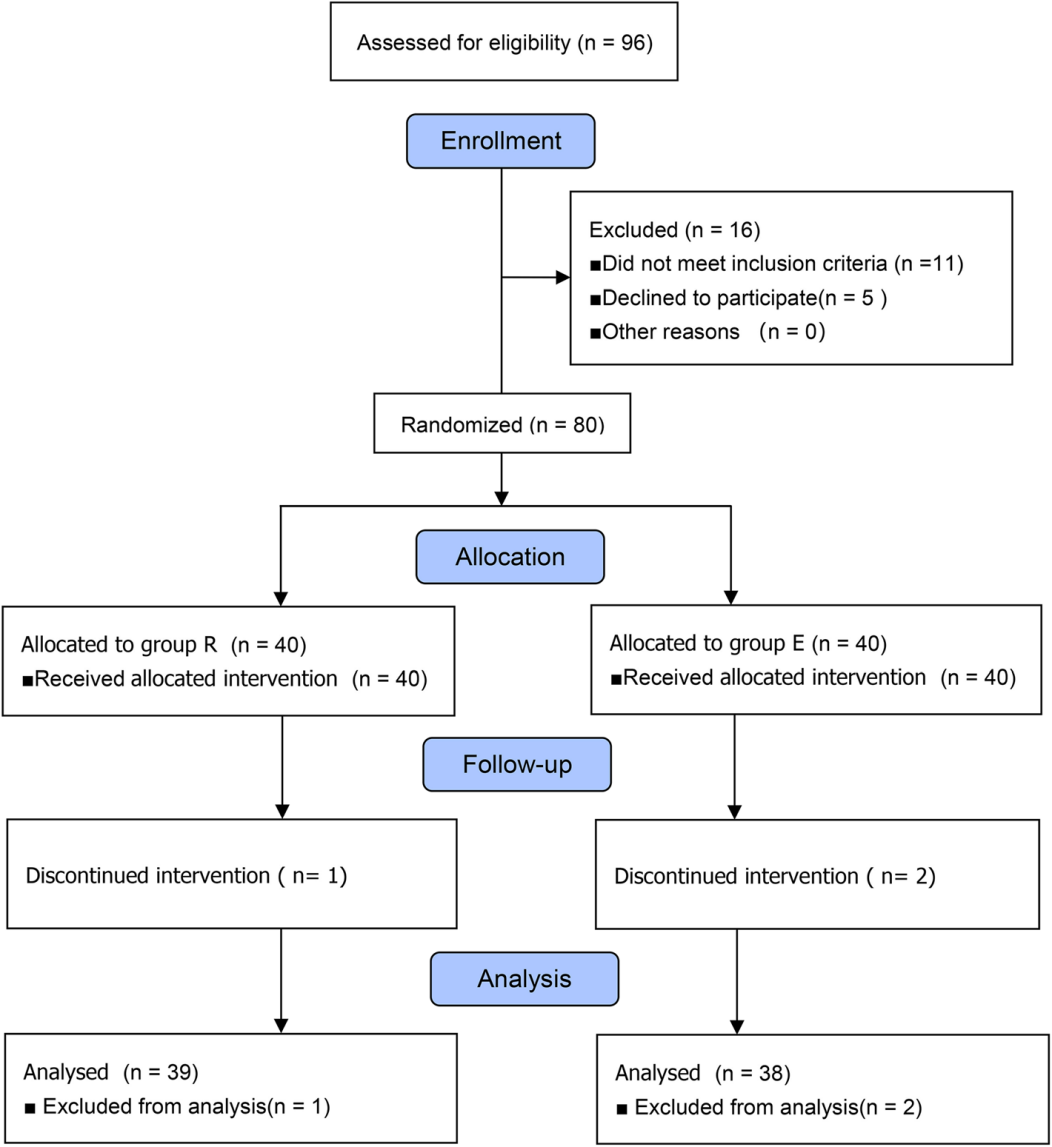
Consort flow diagram

A total of 80 patients were enrolled in the current study, and three patients were excluded from data analysis because epidural was converted to general anesthesia. Thus, the final analyses included 39 patients in group R and 38 patients in group E (Fig. 1). No differences were found in the patients’ baseline characteristics between the two groups (*p >* 0.05) (Table 1).

**Table 1:** Comparison of parturients’ baseline characteristics

	Group R (*n* = 39)	Group E (*n* = 38)	*P* value
Age, years	30.8 ± 3.4	30.3 ± 2.6	0.541
BMI, kg/m^2^	26.7 ± 2.2	27.4 ± 2.0	0.185
ASA, I/II	37/2	36/2	1.000
Pregnancy time, weeks	38.9 ± 0.8	38.9 ± 0.9	0.849
Surgery duration, min	41.7 ± 10.6	46.2 ± 13.9	0.111

Data are expressed as mean ± standard deviation, unless otherwise indicated

BMI = body mass index, ASA = American Society of Anesthesiologists

There were no differences in anesthesia spread levels before surgery and at the end of operation between the two groups (both *p >* 0.05). Compared with group R, the ropivacaine dosage was significantly increased in group E (*p* <  0.001). The usage of remifentanil in group R was 169 ± 14.2 μg. The numbers of patients with ketamine administration and repeat oxytocin administration did not differ between the two groups (both *p* > 0.05) (Table 2).

**Table 2:** Information regarding intraoperative anesthetic drugs and oxytocin usage

	Group R (n = 39)	Group E (n = 38)	*P* value
Anesthesia level, segment			
Before surgery	17.1 ± 0.4	17.2 ± 0.5	0.572
At the end of the surgery	15.9 ± 0.4	16.1 ± 0.5	0.161
Ropivacaine dosage, ml	16.8 ± 0.4^*^	18.0 ± 1.4	< 0.001
Remifentanil dosage, μg	169.2 ± 14.2	0	< 0.001
Ketamine, n	1	4	0.340
Repeat oxytocin administration, n	6	7	0.959
Blood loss during surgery, ml	308 ± 62	311 ± 109	0.888

Data are expressed as mean ± standard deviation or n

^*^Statistically significant difference between groups according to independent-sample Student’s t-tests

Compared with group E, the comfort score was significantly higher in parturients in group R (9.1 ± 1.0 vs. 7.5 ± 1.3, *p < 0.001*), and the maximum VAS score was significantly lower in parturients in group R (1.8 ± 1.2 vs. 4.1 ± 1.0, *p < 0.001*). Compared with group E, the VAS score was significantly lower in group R at T_1_ to T_4_ (all *p* < 0.001). Compared with group E, the incidence of VAS scores ≥4 was reduced in group R (65.8% vs. 12.8%, *p* < 0.001). Compared with group E, the Ramsay score was significantly higher at T_1_, T_2_, T_3,_ and T_4_ in group R (all *p* < 0.001) (Table 3).

**Table 3:** Comparison of Ramsay score between the two groups at T_0_ to T_4_

	Group R (n = 39)	Group E (n = 38)	*P* value
T_0_	1.92 ± 0.27	1.89 ± 0.31	0.670
T_1_	2.18 ± 0.39^*^	1.87 ± 0.34	< 0.001
T_2_	2.26 ± 0.50^*^	1.84 ± 0.37	< 0.001
T_3_	2.56 ± 0.79^*^	1.84 ± 0.37	< 0.001
T_4_	2.71 ± 0.82^*^	1.74 ± 0.45	< 0.001

Data are expressed as mean ± standard deviation or n. T_0_: before anesthesia, T_1_: skin incision, T_2_: delivery of baby, T_3_:uterine suture, T_4_: intraoperative traction

*Statistically significant difference between groups according to independent-sample Student’s t-tests

**Fig. 2:**
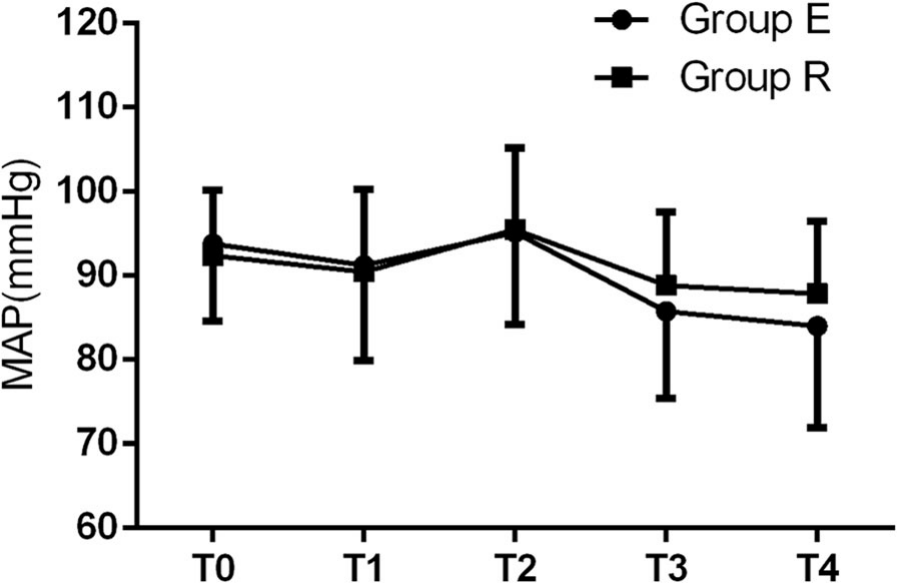
Comparison of mean arterial pressure (MAP) between the two groups at T_0_ to T_4_. There was no significant difference in MAP between the two groups at T_0_ to T_4_ (all *p* > 0.05). T_0_: before anesthesia; T_1_: skin incision; T_2_: delivery of baby; T_3_: uterine suture; T_4_: intraoperative traction

**Fig. 3:**
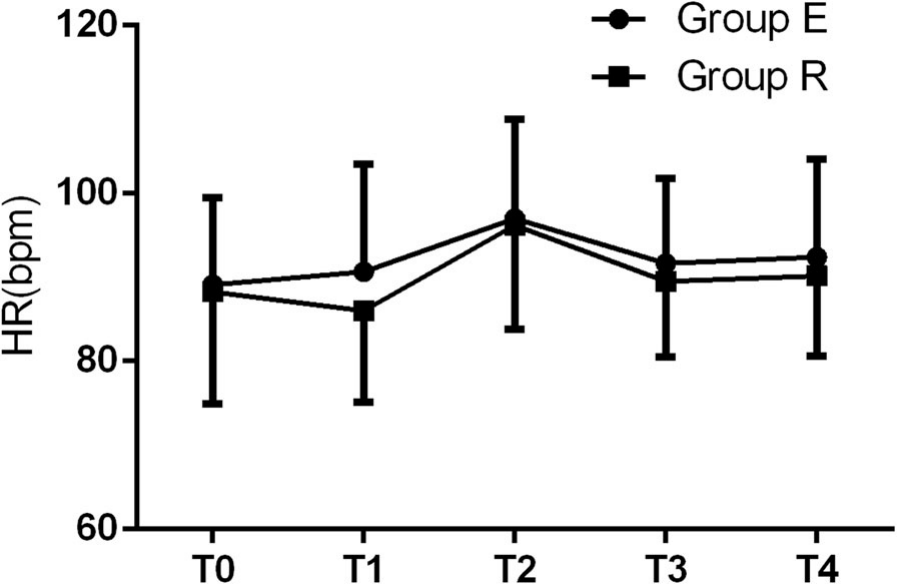
Comparison of heart rate (HR) between the two groups at T_0_ to T_4_. There was no significant difference in HR between the two groups at T_0_ to T_4_ (all *p* > 0.05). T_0_: before anesthesia; T_1_: skin incision; T_2_: delivery of baby; T_3_: uterine suture; T_4_: intraoperative traction

**Fig. 4:**
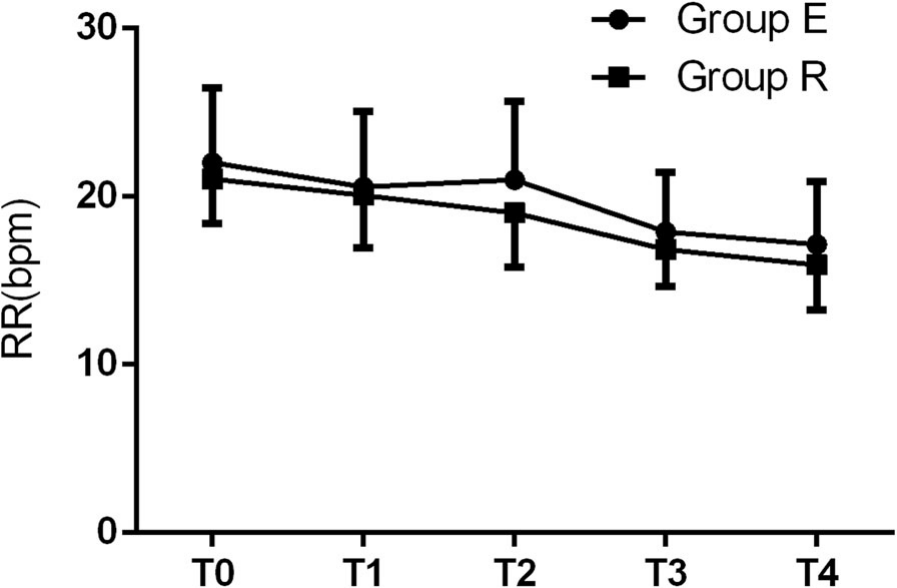
Comparison of respiratory rate (RR) between the two groups at T_0_ to T_4_. There was no significant difference in RR between the two groups at T_0_ to T_4_ (all *p* > 0.05).T_0_: before anesthesia;T_1_: skin incision; T_2_: delivery of baby; T_3_: uterine suture; T_4_: intraoperative traction

There were no significant differences in MAP, HR, or RR between the two groups at T_0_ to T_4_ (all *p* > 0.05) (Figs. 2, 3 and 4). Adverse reactions did not differ between the two groups during surgery (all *p* > 0.05) (Table 4). The number of neonatal resuscitations, pH value of neonatal umbilical arterial blood, and Apgar scores at 1 and 5 min after birth were not different between the two groups (all *p* > 0.05) (Table 5).

**Table 4:** Information regarding adverse reactions

	Group R (n = 39)	Group E (n = 38)	*P* value
Bradycardia, n	1	0	1.000
Hypotension, n	0	4	0.117
Respiratory depression, n	2	0	0.485
Nausea and vomiting, n	1	5	0.191

Data are expressed as n

**Table 5:** Neonatal-related information after delivery

	Group R (n = 39)	Group E (n = 38)	*P* value
Neonatal resuscitation, n	2	2	1.000
pH value of umbilical arterial blood	7.376 ± 0.024	7.380 ± 0.023	0.481
Apgar score			
1 min	9.72 ± 0.60	9.58 ± 0.64	0.331
5 min	9.92 ± 0.27	9.92 ± 0.27	0.974

Data are expressed as mean ± standard deviation or n

PH = pondus hydrogenii

## Discussion

The primary finding of this study was that intravenous infusion of remifentanil could significantly improve the experience of parturients undergoing epidural anesthesia during repeated cesarean section. Visceral pain was relieved during surgery without noticeable maternal or neonatal adverse effects.

In the current study, the comfort scores during surgery were designed as the primary outcome measure of the study. One end of the scores scale is marked as “the most uncomfortable”, the other end is marked as “the most comfortable”, and there is a scale of 0 to 10 cm on the back of the scale.

Previous studies have shown that the incidence rate of visceral pain ranged from 10 to 50% in parturients undergoing epidural anesthesia [4, 13]. In the current study, approximately 65.8% parturients experienced pain (VAS scores≥4) during surgery without remifentanil, which was an apparent increase compared with the rate in previous studies [4, 13] because the subjects were undergoing repeated cesarean section. Indeed, even if the sensory block plane reaches T4, some parturients would experience some degree of visceral discomfort [14]. This maybe because visceral pain is primarily transmitted through unmyelinated C fibers; although the level of sensory block in epidural anesthesia reaches T4, C fibers are not completely blocked. Opioids can inhibit C fibers, in addition to their central analgesic effects [15]. Thus, the parturient who received remifentanil experienced lower visceral pain, such that their comfort scores increased. It is worth noting that ketamine administration dose did not differ from the two groups even though the VAS was lower in parturients with remifentanil. It was because some parturients thought that it was unacceptable using ketamine during repeated cesarean section.

A previous study showed that 0.1 μg·kg^− 1^·min^− 1^ remifentanil could provide effective analgesia during local anesthesia with little influence on respiration and hemodynamics in a general patient population [16]. In the current study, 0.05 μg·kg^− 1^·min^− 1^ remifentanil was administered; we also found that it showed little influence on respiration and hemodynamics in parturients. Although two cases of respiratory depression were found in parturients with remifentanil, it only manifested in the decline of respiratory rate. After reducing the speed of remifentanil infusion and awaken the patient, the respiratory rate returns to normal. Notably, parturients with epidural anesthesia alone may experience greater hypotension because larger doses of ropivacaine were used. Avramov et al. [16] and Kan et al. [17] reported that remifentanil could also provide a degree of sedation, which improved patient comfort levels in the current study. In obstetric surgery, it is important to determine whether administered drugs affect uterine contractions. The present study showed that intravenously administered low-dose remifentanil had a minimal effect on uterine contraction, based on the requirement for repeated usage of oxytocin and the blood loss during surgery.

Kan et al. [17] reported that remifentanil can cross the placenta, and that it appears to be rapidly metabolized, redistributed, or both, without neonatal adverse effects. Lee et al. [18] also found that remifentanil can be safely used for vaginal delivery. In the present study, as reported in previous studies, no neonatal adverse effects were observed, including changes in rates of neonatal resuscitation, the pH value of neonatal umbilical arterial blood, and Apgar scores. Van de Velde et al. [19] reported that a 0.50 μg/kg intravenous bolus of remifentanil induction dose, followed by a continuous infusion of 0.20 μg·kg^− 1^·min^− 1^, caused partial neonatal depression, and required brief mask-assisted ventilation. Noskova et al. [20] reported that a bolus of 1 μg/kg remifentanil before induction of general anesthesia decreased Apgar scores at 1 min after cesarean delivery, although the clinical symptoms were short. These observations were not found in the current study, because the dosage of remifentanil injection was lower than in the prior studies [19, 20].

In this study, four neonates were needed resuscitation. In parturients with remifentanil, the reasons of resuscitation were umbilical cord around neck and the long delivery time of the fetus (from skin cutting to fetal delivery). In parturients without remifentanil, the reasons of resuscitation were umbilical cord around neck and amniotic fluid III degree. Although none of neonatal resuscitation was caused by remifentanil, we should consider the potential risks of using remifentanil in the clinic, decrease the dose of remifentanil and careful monitoring. Thus, low dosage of remifentanil is recommended for parturients during repeated cesarean section.

The current study has some limitations. First, previous studies have shown that epidural opioid administration may relieve visceral pain under epidural anesthesia in parturients [3, 21], but we did not compare the effects of intravenous remifentanil and epidural opioid on visceral pain relief. Second, only 0.05 μg·kg^− 1^·min^− 1^ remifentanil was used in the current study; whether this is the optimum dose requires further research. Third, neonatal resuscitation during cesarean section would occur. In order to facilitate the obstetrician to analyze the reasons for the decrease in Apgar scores, the study was not designed to be a double-blinded study, and it was hard to exclude the patients’ perception, which would increase the bias.

The present study demonstrated that, in parturients undergoing repeated cesarean section, continuous intravenous infusion of low-dose remifentanil could significantly enhance the parturient experience during epidural anesthesia, without obvious maternal or neonatal adverse effects.

## Abbreviations

ASA: American Society of Anesthesiologists
BMI: Body mass index
HR: Heart rate
MAP: Mean arterial pressure
RR: Respiratory rate
SBP: Systolic blood pressure
SpO_2_: Saturation of pulse oxygen
VAS: Visual analogue scale
